# De-Sexualizing Partner Notification: A Qualitative Study on Chinese Young Adults with Chlamydia

**DOI:** 10.3390/ijerph18084032

**Published:** 2021-04-12

**Authors:** Bobo H. P. Lau, Lucia Liu, Celia H. Y. Chan, Cecilia L. W. Chan, Jason J. Ong, Eleanor Holroyd, William C. W. Wong

**Affiliations:** 1 Department of Counselling & Psychology, Hong Kong Shue Yan University, Hong Kong; hplau@hksyu.edu; 2 Ho Yuk Ching Educational Psychology Service Centre, Tung Wah Group of Hospitals, Hong Kong; lucialiu60@gmail.com; 3 Department of Social Work and Social Administration, and Centre on Behavioral Health, The University of Hong Kong, Hong Kong; chancelia@hku.hk (C.H.Y.C.); cecichan@hku.hk (C.L.W.C.); 4 Central Clinical School, Monash University, Melbourne, VIC 3800, Australia; doctorjasonong@gmail.com; 5 Department of Nursing, Auckland University of Technology, Auckland 1142, New Zealand; eleanor.holroyd@aut.ac.nz; 6 Department of Family Medicine and Primary Care, Li Ka Shing Faculty of Medicine, The University of Hong Kong, Hong Kong

**Keywords:** chlamydia, sexually transmitted infection, partner notification, stigma, Chinese

## Abstract

Background: Chlamydia is common amongst the sexually active population in Hong Kong. As most cases are asymptomatic, partner notification may be helpful in controlling chlamydia. This study examined attitudes towards partner notification for chlamydia among Hong Kong Chinese youths in order to inform a culturally appropriate, patient-empowering sexual health service. Methods: Sixteen individuals (aged 20 to 31) who received a confirmed diagnosis of chlamydia within the previous twelve months of data collection were recruited from two community-based organizations between June and December 2017. Semi-structured individual interviews were conducted by a health psychologist. Results: Nine participants notified a total of eleven current and ex-partners. Seven participants did not notify their sexual partner(s). Our findings revealed how participants struggled with the discrediting sexual aspect of their infection, and how de-sexualizing the infection and selected disclosure facilitated partner notification and social acceptance. Perceived stigma regarding chlamydia however did not dissipate with their disclosure. Participants did not perceive lasting impact of chlamydia on their well-being as they thought they have much control over whether and how to disclose to their (future) partners. All participants agreed there was a pressing need to raise public awareness on this silent but highly prevalent sexually transmitted infection. Conclusions: Our findings illustrate the complex struggle behind communicating about chlamydia to one’s sexual partner and how strategizing the disclosure process served to circumvent embarrassment and foster testing of sexual partners.

## 1. Introduction

Chlamydia is the most common bacterial sexually transmitted infection (STI) worldwide, with a global prevalence of 3.8% and 2.7% among females and males aged 15 to 49 years [1]. The prevalence of chlamydia in China (e.g., 2.6% in women, 2.1% in men from Parish et al. [2]; 4.12% in women from Luo et al. [3]) is comparable to international estimates. In a population-based representative sample, our team found high prevalence of chlamydia among sub-populations in Hong Kong i.e., 5.8% in younger (18–26 years old) sexually-active women; 4.8% in sexually-active men; and 4.1% in older (40–49 years old) sexually-active women [4]. Chlamydia is a significant public health challenge because of the associations of repeated infections with infertility and adverse pregnancy outcomes as well as the co-occurrence with other STIs [5]. As chlamydia is often asymptomatic, partner notification is critical for preventing re-infection of the index patient and reducing ongoing transmission in the community [6]. A cost-effectiveness analysis shows that increasing partner notification from 0.4 to 0.8 partner per index case can be six times more cost-effective than expanding coverage of chlamydia screening from 8% to 24% in the UK [7]. While partner notification is important for controlling the spread of the infection, many individuals find it difficult and daunting to inform anyone of their diagnosis [8,9,10,11].

Historically, sex and the expression of sexuality have been censored by Confucianism in the Chinese culture [12,13]. In Confucianism, the repressive attitude on sex aims at enforcing chastity. “Good sex” means sexual intercourse for the sole purpose of producing a healthy male heir for the paternal family, while sex that occurs out of wedlock (e.g., premarital sex, homosexuality, commercial sex) is stigmatized and reprimanded. These conservative attitudes toward sex were maintained until the end of 20th century when international exchange became more common. The first wave of change occurred with the medicalization of sex by focusing on the biological aspects of male and female sexual function and sex-related pathology in married couples. In the next wave of change, sex was seen as a social problem studied by social scientists who were interested in divorce, prostitution, and single women [14]. Recently, there has been an increasing focus on personal intimacy and relational equality in sex, and that sex as a subject matter was no longer as medicalized and pathologized [15]. For instance, premarital sex and extra-marital sex have become more widely accepted as individual preferences and choices among young Chinese people, as long as sex is consensual and the partners are in a romantic relationship [13].

However, this de-traditionalization is far from complete and liberalization occurred mostly “inside the bedroom” (i.e., the private sphere) rather than in the public discourse. In Zheng et al. [16], 15% of respondents in an interview study on extra-marital sex regarded sex as a private matter that should not be discussed with a stranger and were reluctant to answer some related questions despite being framed in a depersonalized way. Furthermore, the greater personal freedom to experiment with sex also came with greater attribution to individual responsibility when things go wrong. Take premarital sex as an example; those who approve of it cited the changing social climate as their reason for approval. However, the act is only morally approved if it is legal and does not harm the interest of anyone. The emphasis on responsibility and prevention of harm, rather than rights and pleasure, is still coherent with the Confucian mores on social relationships.

The Disclosure Process Model (DPM) [17] is well-suited for conceptualizing partner notification in the context of a concealable stigmatized health condition, like chlamydia. Accordingly, partner notification begins with identifying the antecedent goals of partner disclosure (e.g., a positive outcome such as an enhanced relationship or preventing a negative outcome such as stigma), followed by the disclosure event which is characterized by its content (i.e., what information, and when, how, and to whom to disclose) as well as the reaction of the sexual partner(s). The individual, relational, and socio-contextual impacts of the disclosure are mediated by processes such as the relief of inhibition as well as changes in social support and social information.

During partner notification, Balfe and Brugha [18] observed that patients spend considerable efforts to suppress the discrediting characteristics of their STIs (e.g., sex partners, coital behaviors). In the Chinese context, Confucian mores and social norms may generate a strong stigma against pathologies related to sex and therefore hinder honest disclosure and discussion about high-risk sexual behaviors. Liu et al. found Chinese men with an STI who felt stigmatized were 58% less likely to notify their spouse of their infection [19]. Suppression of these discrediting details might be achieved through de-sexualizing the infection, defined as “downplaying or hiding information about sexual transmission and the importance of high-risk sexual behavior”. This strategy has been observed for boosting public acceptance of human papillomavirus (HPV) and Hepatitis B vaccination [20,21].

Most evidence of partner notification of chlamydia comes from studies conducted in predominantly European-American societies, such as in Australia [22], the UK [9], and the USA [23]. Few studies on partner notification have been conducted for ethnic Chinese. It is not clear how the practice of partner notification may differ in cultures with more conservative attitudes toward sex. Therefore, this study examined attitudes towards partner notification for chlamydia among Hong Kong Chinese.

## 2. Materials and Methods

### 2.1. Sampling and Recruitment

Individuals aged 16 years or above who received a confirmed diagnosis of chlamydia within the previous twelve months were recruited. Individuals who were not ethnically Chinese and could not communicate in Cantonese; not residing in Hong Kong; or, had a self-reported history of severe psychiatric conditions (e.g., schizophrenia, psychosis) were excluded.

Eligible participants were recruited from two community-based organizations (CBOs)—AIDS Concern and a Facebook sexual health education platform known as StickyRiceLove—between June and December 2017. AIDS Concern runs a free testing and counselling service for HIV, syphilis, gonorrhea, and chlamydia. The staff examined their client database for eligible participants and approached 49 individuals; 31 declined to participate and three agreed to participate but did not show up due to time constraints. Fifteen participants (15/49 = 30.6%) were recruited by AIDS Concern, and one additional participant was recruited from StickyRiceLove.

### 2.2. Procedure

Semi-structured individual face-to-face interviews were conducted either at the interview room of AIDS Concern or a private counselling room of the institution of the corresponding author. After written consent was signed, the interviews were audiotaped. The interviews lasted between 45 min to 2.5 h. All interviews were conducted in Cantonese by the first author, a female bilingual researcher with a background in health psychology. Each participant was reimbursed 200 HKD (1 USD = 7.8 HKD). Data collection ceased when data saturation was reached.

### 2.3. Interview Schedule

Our research team, comprising a professor in sexual health and professors and researchers in reproductive health and medical social work, developed the semi-structured interview guide based on the Disclosure Process Model (DPM) [17] that covered psychological reactions and perceived relational repercussions of chlamydia infection, testing and treatment experience, partner notification, and recommendations for healthcare professionals. The partner notification process was divided into goals and reasons for disclosure (or non-disclosure), information provided during the disclosure, context of the disclosure event, reactions of the confidant, and perceived impact on the relationship between the participant and their sexual partner(s). (See Supplementary Material 1 for the interview schedule).

### 2.4. Data Analysis

All interviews were transcribed in Cantonese with each transcription checked by two coders for accuracy. Thematic analysis was conducted using NVivo (version 11) for data management. First, the two coders familiarized themselves with the data through reading and re-reading. Then, codes were generated based on the DPM and literature about chlamydia partner notification [8,9,17,18,22]. New codes emerged when the existing codes failed to represent the content of the data unit. After double-coding and attaining agreement through iterative discussions by the two coders, potential themes were generated through re-examining the codes. The themes were then refined and formally defined through discussions among the research team.

## 3. Results

Sixteen participants took part in the study. Thirteen were female aged between 20 to 31 years (Table 1). Nine reported being in a stable relationship and six were single at the time of data collection. One participant, who had a son, was undergoing divorce and the rest were childless. All but one was heterosexual. All reported that it was the first time they had chlamydia and had relatively few sexual partners (43.1% had 0 to 1 partner in the previous year).

Nine participants (8 female, 1 male) notified eleven partners, while seven participants did not notify their sexual partner(s). Five participants notified their current partners, two their ex-partners, and two notified both current and ex-partners. Eventually, only two sexual partners sought chlamydia testing with one informing the participant of his negative test result. In two cases, the participants’ current partners were being managed for STIs, therefore, the participants also went for testing and informed their current partners of their test results.

### 3.1. Disclosure as a Difficult Decision But a Moral Responsibility

Participants found partner notification “embarrassing and difficult”. They worried about the potential negative reactions, including rejection and contempt, from their partners as well as the possibility of being stigmatized and gossiped about. Participants who eventually notified their partners did so out of the moral obligation to “tell the truth” and for the perceived health benefits of their current and previous partners with whom they had been close with, as well as the partners of their partners whom they would assume to be infected unknowingly.

First, I want her (ex-girlfriend) to be well. Second, I don’t want others to have it. (Participant 007; male; disclosed).

Anticipating negative reactions from the sexual partners discouraged disclosure. These participants worried that their current relationship would be jeopardized because their sexual partner may accuse them of being promiscuous. Being out of contact with the casual partner or being in a conflictual relationship provided valid reasons for non-disclosure, as the participants thought the communication channel was simply absent. The absence of symptoms also increased the difficulty of notification, as the sexual partner may disbelieve or hold the participant single-handedly responsible for the infection as nobody knows who was the “source”.

I think this (chlamydia) is a disgrace. [Interviewer: But it might not be not your fault] But I think he (a sexual partner) anyway will mind. Likewise, if my partner tells me he has a STI, although it’s none of his fault, I won’t be all comfortable. (Participant 005; female; not disclosed)

I think I may send him (a sexual partner) a relevant website and ask what he thinks about it. If his reaction is unpleasant and it’s likely that he will gossip about me, I won’t disclose. If his reaction is OK and normal, I think there will be a better chance I will disclose. (Participant 015; female; disclosed)

### 3.2. Embarrassment of Disclosure Reduced with the Belief in a Non-Sexual Cause of One’s Infection

Chlamydia is an unfamiliar infection among the Hong Kong public. Participants remarked they have either not heard of this term or had received contradictory and confusing information from the media prior to their diagnosis. Several participants firmly believed their infection was due to poor personal hygiene (e.g., dirty toilet seats, sharing contaminated towels) rather than unprotected sex, even though they now understood the infection is sexually transmissible. Some considered chlamydia as merely a urinary tract infection (UTI), but their medical consultations were not helpful in dispelling this misconception. For them, chlamydia could be caused by non-sexual means but transmissible to their partners through sex. Partner disclosure was therefore a natural consequence of a rather benign diagnosis in order to protect their current partners. Hence, for these participants, difficulty and embarrassment were neither experienced nor anticipated as the infection was not considered as associated with condomless sex.

Interviewer: Okay. Was he worried about your condition?

Participant: To my knowledge, not really. I think this (chlamydia) is just because of a dirty toilet.

Interviewer: Did he ever thought about it might not be because of the toilet? Do you think he had ever suspected anything related to sex?

Participant: To my understanding, no.

Interviewer: So, are you two still together?

Participant: Yes. (Participant 014; female; disclosed)

### 3.3. Strategizing Disclosure by De-Sexualizing the Mode of Transmission and Testing

Most disclosure was conducted via instant messaging (e.g., WhatsApp) or telephone to avoid the embarrassment from face-to-face interactions usually shortly after the diagnosis. Disclosure was conducted in a “matter of fact” manner through direct information-giving. As the disclosure was pragmatically geared toward encouraging chlamydia testing for their sexual partner(s), the most common information provided was where the testing can be done—only with more trusting relationships was more detailed information disclosed. In order to minimize confusion and embarrassment, participants tended not to mention the term “chlamydia”, nor its mode of transmission, treatment, and (the lack of) symptoms.

I wrote a message on WhatsApp which said I have no bad intentions, but I want her (ex-girlfriend) to be careful (in future sex), it is for her benefits. I told her the treatment method, the antibiotics and suggested her to go and check with a doctor. (Participant 007; male; disclosed)

Reflecting on their experience of disclosure (and non-disclosure), several strategies were suggested, with some having been employed and led to positive results (Table 2). Notably, participants disguised their STI test as a gynecological examination or general body check, denied any sexual disloyalty or risky sexual behaviors, or fabricated stories about non-sexual means of transmission (e.g., contaminated underwear and towels, dirty swimming pools). Some participants suggested to reframe chlamydia as an UTI that is sexually transmissible in order to encourage testing, re-testing, and treatment.

Our society always regard sex as such a negative thing and STI as a severe illness and is very negative. Why not call it (chlamydia) an UTI? For UTI, it might be difficult to tell (the cause), it could have come from a dirty toilet in a mall. But right now, the STI label makes it a very negative thing, but the experience of the infection itself is not too negative. (Participant 014; female; disclosed)

### 3.4. Lack of Relief Despite a Positive Response from Their Sexual Partner(s)

In six of the disclosure to current or ex-partners, participants reported that their partner(s) were generally supportive despite their initial bewilderment. Anger and sadness from their partner(s) were reported in cases where they blamed the participants and their ex-partners for their high-risk sexual behaviors, yet these negative emotions quickly dissipated. Most partners reasoned a person should not be blamed for transmitting an infection when he/she did not know they had it. Instead of feeling relieved, the participants continued to worry about unforeseen negative impacts on their (or future) relationships, expressed regret over not having practiced safe sex, or even felt guilty about potentially infecting their current partner. Perceived stigma regarding the STI tended not to dissipate with the disclosure.

We had some conflicts. He (current boyfriend) said he thought I was a nice girl, he didn’t use the word “clean” to describe me, but he thought I was, how should I put it, safe, not possessing anything bad, etc. He said that was why he didn’t use a condom with me. I thought I didn’t know I’ve been infected too, how dare he say something so hurtful. (Participant 013; female; disclosed)

In cases where participants believed and conveyed a non-sexual cause for their infection, they reported that there was only a slightly negative reaction from their partner(s). For these partners, chlamydia was perceived as an unfortunate infection, just like a flu, with no impact on their relationship. In disclosure to ex-partners with whom the participants had little recent contact, sexual partners tended to be apathetic to the notification.

### 3.5. Changes in Sexual Practices

Changes in sexual practices, including abstaining from vaginal intercourse while taking the prescribed antibiotics, using male condoms, reducing the number of sexual partners, and showering before sex were reported in both disclosing and non-disclosing participants. Similar changes were found in those who had believed in non-sexual causes of their STI as one of the many ways to improve their general hygiene. Among participants who put forward a non-sexual cause for their infection during partner disclosure, the mere act of partner disclosure paradoxically confirmed the sexually transmissible nature of the infection and provided a valid ground for urging these changes in sexual practice with their sexual partner(s).

### 3.6. Impacts on Personal and Relational Well-Being

Participants neither experienced nor anticipated a lasting impact from chlamydia on their personal and relational well-being, as the infection was understood as being easily treatable with a short course of antibiotics. Their relief also came from perceiving control on whether and how to notify their (future) partners through carefully assessing the relational context at hand. Apart from pledging to use condoms for sex in the future and re-test when changing a new partner or after condomless sex, participants took the opportunity of struggling with partner notification to review their assumptions about STIs in general. Regardless of the disclosure and the perceived cause for their infection, all participants agreed there was a pressing need to raise public awareness and de-stigmatize this silent but prevalent STI.

However, they remarked that chlamydia was “different” from the STIs they could readily recall. Chlamydia was seen as “less scary” as it did not frequently manifest with stereotypical, visually striking symptoms (e.g., genital ulcers, unsightly genital discharge, rashes) and can be easily treated. This led to some participants suggesting reframing chlamydia as an UTI that can be sexually transmitted—a concept that may render less personal responsibility and stigma to the index patient, and encourages honest disclosure and testing.

## 4. Discussion

**Figure 1:**
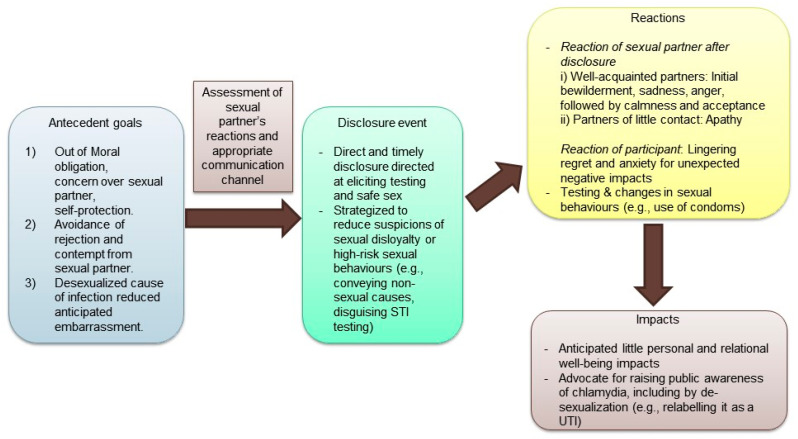
Model on partner disclosure of chlamydia in Hong Kong Chinese.

Despite the recent liberalization of sex in the private sphere that has led to greater experimentation [12,16], sex and STIs remain a taboo for open discussion. Under such a socio-cultural backdrop, this study is the first to examine the process of partner notification of chlamydia among young Hong Kong Chinese. Our participants used a range of de-sexualizing strategies to circumvent the undesirable experiences when disclosing their diagnosis to their partners (see Figure 1).

Our study was guided by the DPM [17] that dissected partner notification into antecedent goals, disclosure event and their sexual partner’s reactions, and mediating processes and impacts. In similar studies conducted internationally [8,11], participants tended to disclose out of a sense of the moral obligation to safeguard the health of their partners, even though they worried about the negative repercussions. As noted by Balfe and Brugha [18], the disclosure could be strategized to avoid the undesirable impact arising from social disapproval of STIs and from suspicion of discrediting sexual behavior and/or sexual disloyalty. Most participants disclosed through non-face-to-face communication in order to reduce embarrassment and confrontation. The disclosure events were carefully staged to elicit pragmatic actions from their sexual partner(s), including encouraging testing and sex with condoms. As a result of the participants’ meticulous calculation of their partner’s reactions and strategized disclosure, all participants who disclosed reported minimal long-lasting negative impact on their relationships, with the exception of their partner’s initial bewilderment, anger, and/or sadness. These findings concur with that of a Dutch study which documented minimal unfavorable relational impact post-disclosure as the participants tended to have carefully planned to whom and how to disclose [24]. In the current study, however, participants remained anxious and regretful despite their disclosure. It is possible that since their disclosure was often partial, discrediting details (e.g., past casual sex) were not “granted acceptance” by their sexual partner(s), and these details remained an uncomfortable secret for these participants.

In contrast with the Western literature, we found our participants were keen on de-sexualizing chlamydia. The exclusivity of condomless sex as the cause for chlamydia were questioned by participants, while non-sexual causes were believed or fabricated by the participants and conveyed to their sexual partners. Other de-sexualizing strategies, such as suggesting the tests were just general health examinations and directing the disclosure conversation to desired action rather than past sexual behaviors, were undertaken to mask any embarrassing sexual behaviors. Participants appeared to accept the infection is transmissible to their partners through sex but were uncomfortable with their infection originating from a sexual intercourse that deviates from the traditional expectations of “good sex”, which is between two healthy and informed individuals in wedlock or a serious romantic relationship. Individuals who practiced “bad sex” were usually linked to a person’s character as “lewd” [25]. Therefore, de-sexualization could be a strategy to avoid being morally judged. This strategy may be possible in Hong Kong’s context, as the Chinese term for chlamydia, which signifies some sort of external body that can cause an infection (yi yuan ti), is unbeknownst to the community nor self-explanatory. The lack of symptoms, easy treatment, and invisible impact on one’s long-term fertility of chlamydia rendered ambiguity in its terminology, and so its pathology, unchallenged and unaddressed in the public discourse. Flawed information, such as confusion with UTI and non-sexual causes has slipped in and filled the vacuum of knowledge on this stigmatized infection.

While de-sexualization may take the heat off the disclosure of stigmatizing sexual behaviors during partner notification, it promotes inaccurate health information and reinforces social disapproval of STIs. Thus, dispelling stigmatization and misconceptions about chlamydia through education is an essential first step. We do not agree that reframing chlamydia as an UTI rather than a STI would be helpful. Instead, we recommend public education to take advantage of the conveniently treatable nature of chlamydia to rectify the overwhelmingly negative image of this STI. Informing the public about the high prevalence of chlamydia may also enhance the perceived value of sex with condoms. Provision of accessible age-based instead of behavior-based screening may de-stigmatize and increase acceptance of testing [26]. A review concluded that providing information and counselling to assist the index patients to notify their partners yielded comparable efficacy in preventing re-infection compared to expedited partner therapy [27]. Counselling to support participants to communicate their diagnosis and handle the relational aftermaths and perceived stigma should be made available to individuals who have tested positive in order to enhance their self-efficacy of partner notification. As the participants have mentioned, it took them courage to disclose such a stigmatizing diagnosis to their partners, and they did so out of their care, concern, and responsibility to their sexual partner(s). Therefore, the love, altruism, and goodwill between the sexual partner and the index patient could be emphasized when encouraging partner disclosure. Counsellors could also advise the appropriate means of disclosure (e.g., timing, channels) based on the context of clients’ relationships.

### Strength and Limitations of the Study

In contrast with previous studies that recruited key populations (e.g., sex workers, men who have sex with men), this study did not limit the recruitment to populations with elevated behavioral risks. Instead, a group of young adults with relatively low risk and infrequent exposure to high-risk contexts were recruited from the community. Thus, these narratives may be more reflective of the general public’s views and are informative for healthcare professionals to educate the public about this silent but prevalent STI. Based on the DPM, our study organized partner notification into stages, and our findings may guide future studies to examine the causal relationships of constructs at different stages of the partner notification journey.

Our study had several limitations. We relied on a relatively small sample accrued over a long period of time. This actually reflects the stigma surrounding STIs and the resultant unwillingness to discuss them. There was also insufficient representation from males, homosexual individuals, and people of younger or older ages, which would have added useful insights. The majority of our participants were women, and the small sample prevented any gender analysis, which may reveal gender differences in perceived stigma and partner disclosure. We urge future studies to examine the gender differences in STI disclosure, understanding that expectations regarding promiscuity and sex-related shame may affect males and females differently. Our participants, despite being recruited from the community, could have held more open attitudes toward sex and STIs compared to those who declined our invitation or had never tested despite having condomless sex. In fact, to preserve the rapport between the referrer and the potential participants, we did not log the reasons for refusal other than time constraints. Due to the sensitive nature of STIs, recruitment outside AIDS Concern and StickyRiceLove was almost impossible. We attempted to obtain referrals from a private venereologist throughout the data collection period but did not receive any referrals. The asymptomatic nature of the infection might have also hindered testing, adding difficulties to our recruitment.

## 5. Conclusions

This is the first study to explore partner notification of chlamydia infection in a Chinese non-high-risk population sample. Through interviews with sixteen local young adults who had a recent chlamydia diagnosis, our findings reveal how participants struggled with the discrediting sex-related aspects of chlamydia and how de-sexualizing their infection was perceived to facilitate partner notification and foster social acceptance. These first-hand experiences could inform healthcare professionals to devise effective public education and suitable measures that enhance partner notification.

## Figures and Tables

**Table 1:** Sample characteristics (N = 16).

Variable	Category	Frequency (%)
Gender	Male	3 (18.7%)
	Female	13 (81.3%)
Mean age (range)		23.4 (20 to 31) years
Education	Undergraduate or above	10 (62.5%)
	Higher diploma	3 (18.8%)
	Senior secondary	2 (12.5%)
	Junior secondary	1 (6.2%)
Occupation	Student	1 (6.2%)
	Full-time worker	10 (62.5%)
	office administrative	6 (37.5%)
	finance	1 (6.2%)
	design	1 (6.2%)
	healthcare professional	1 (6.2%)
	social work	1 (6.2%)
	Part-time worker	4 (25.0%)
	Housewife	1 (6.2%)
Current relationship	Single	6 (37.5%)
	Stable relationship	9 (56.3%)
	Divorced	1 (6.2%)
Number of sex partners in previous year	0–1	7 (43.8%)
	2–5	8 (50.0%)
	>5	1 (6.2%)
Sexual orientation	Heterosexual	15 (93.8%)
	Homosexual	1 (6.2%)
Previous urinary tract infection (UTI) or	Candidiasis	3 (18.8%)
sexually transmitted infection (STI)	UTI	1 (6.2%)
	Unknown	1 (6.2%)
	No history of UTI or STI	11 (68.8%)

**Table 2:** Strategies suggested by the participants for partner disclosure.

1. Disguise STI testing as gynecological examination or general body check: Maybe I will make up stories about having done a body check, something like those provided by medical insurance, and the check includes a STI test and there I got the results. (Participant 010; female; disclosed)
2. Emphasize the possibility of non-sexual means of transmission (e.g., contaminated underwear, sharing contaminated towels in a trip abroad, dirty toilets): I would say even if you have not had sex, you could be infected, for instance the towels, or the weather is too hot and there wasn’t thorough cleansing. (Participant 007; male; disclosed)
3. Focus the conversation on the desired action (e.g., practice safe sex in the future, testing) rather than the cause or the embarrassing past: I skipped the part related to my one-night stand and only urged him (current partner) to have a check. (Participant 002; female; disclosed)
4. Emphasize sexual loyalty with the current partner: I told him (current partner) directly I have not been fooling around, before I mentioned anything about the test. (Participant 002; female; disclosed)
5. Nominate a distant ex-partner as the source of the infection: Even if he (current partner) kept asking I couldn’t give him an answer. I just randomly mention an ex-partner, he anyway doesn’t know my ex-partners. (Participant 013; female; disclosed)
6. Make comparisons with other more severe STIs (e.g., HIV, syphilis): I asked him (current partner) to guess my diagnosis. He came up with something much scarier. Then I said no and that I had chlamydia. (Interviewer: Was he relieved?) Yes. But this is still a good thing, now he treats chlamydia more seriously. (Participant 015; female; disclosed)
7. Disclose information in manageable bite-size. Do not overwhelm the sexual partner with esoteric medical terms (including the term “chlamydia”): (Interviewer: Did you tell her it is chlamydia?) No, because many people don’t know what chlamydia is. (Participant 007; male; disclosed)
8. Disclose with a comfortable means for conversing private and sensitive topics: I have thought about finding a time to talk about it face-to-face. But I’m afraid I can’t kick off the conversation. (Interviewer: What about not doing it face-to-face?) A phone call then. At least a call to disclose a short version, that is roughly what I have, and then leave the details to the face-to-face meeting. With that phone call to begin with, we won’t be that embarrassed when meeting up. (Participant 013; female; disclosed)

## Data Availability

The data of this study is available via emailing the corresponding author.

## Supplementary Materials

The following are available online at https://www.mdpi.com/article/10.3390/ijerph18084032/s1, Supplementary Material 1. Interview schedule.
