# Supplementary material for: De-Sexualizing Partner Notification: A Qualitative Study on Chinese Young Adults with Chlamydia
Source: Int J Environ Res Public Health. 2021 Apr 12;18(8):4032. doi: 10.3390/ijerph18084032 (PMC8070504; doi:10.3390/ijerph18084032)
Supplement: Supplementary file 1 [file ijerph-18-04032-s001.pdf]

Supplementary Material 1. Interview schedule

Part A: Understanding of chlamydial infection.

1. Can you tell me about your experience with chlamydia?
   Prompts:
   How did you come to attend the clinic?
   What happened to you during the clinic visit?
   What did the doctor tell you?
   What was your expectation with the medical treatment?
   How do you think you got infected?
   How long do you think you have the infection in your body?
   Why do you think it was happening to you?

Part B: Quality of life and sexual health impact of chlamydial infection.

2. How were you and your partner/ significant others impacted by your chlamydia?
   Prompts:
   What were the first reactions after you had received the diagnosis?
   What were the consequences of your chlamydia to your health and daily functioning? How much and why were these consequences important?

   What were the consequences of your chlamydia to your social life? How much and why are these consequences important?
   Who did you blame for / angry at for the infection and why?
   What does chlamydia or STI mean for you as a woman/man?
   What are your worries and concerns now?
   What are the implications on your close relationship? How much and why are these consequences important?
   What does your chlamydia mean to your close relationship?

3. How has your sex life been affected by chlamydia? How much and why are these consequences important?
   Prompts:
   Frequencies of sexual intercourse, sexual behaviors, use of condoms and other contraceptive measures, initiation/fore-play, pleasure, attitudes, overall satisfaction, number of sex partners, use of sex toys, what is done after the intercourse etc.

Part C: Partner notification.

4. Do you have a sexual partner during the time you received the diagnosis?
   4a. Did you tell him/her about your chlamydia?
      If yes : to one partner or other partners?
      i. Why did you tell him/her?
      ii. How did you tell him/ her?
      iii. What were his/her reactions?
      iv. How did you feel after revealing the diagnosis to him/her?
      v. What did the both of you do / are the both of you going to do after knowing the diagnosis?
      vi. In general, do you see any differences in your relationship with the confidant after you have revealed the diagnosis?

If no :

i. Why did you not tell him/her?
ii. What do you think his/her reaction would be if you tell him/her the diagnosis?
iii. How do you feel now?
iv. What are you going to do?
v. In general, do you see any differences in your relationship with him/her compared to before the diagnosis?

5. Will you tell your future partner about your history of chlamydia and why?

Part D: Clinical expectation

6. What can the healthcare professionals do to make your experience easier?
   Prompts:
   Did healthcare professional make you feel bad during the treatment. If yes, would you share your experience?
7. What advice would you give to people who have been diagnosed with chlamydia?
